# Silicate Sawing Sludge Recovery in Thermo Eco-Mortar for Macroporous Plaster

**DOI:** 10.3390/ma13061293

**Published:** 2020-03-13

**Authors:** Lorena Zichella, Paolo Marone, Pasquale Buonanno, Marco D’Amore, Rossana Bellopede

**Affiliations:** 1 Department of Environment, Land and Infrastructure Engineering (DIATI), Politecnico di Torino, 10129 Turin, Italy; rossana.bellopede@polito.it; 2 I.S.I.M.—Istituto Internazionale del Marmo, 20154 Milan, Italy; paolo.marone@stonetechtraining.it (P.M.); m.damore@unina.it (M.D.); 3 Bunker TEK.SP.ED. srl, Casandrino (NA), 80025 Casandrino, Napoli, Italy; pasquale@bunker-teksped.com

**Keywords:** silicate sawing sludge, eco-plaster, waste management, circular economy, waste recovery

## Abstract

The aim of this research was to develop a new eco-product to find a correct recovery of silicate sawing sludge by means of waste management according to European criteria. To reach this goal, a thermal eco-mortar for a macroporous plaster was developed. The main characteristics of a plaster that influence the correct choice of the mortar are good adherence with underlying support, impermeability, thermal and acoustic insulation, mechanical resistance and ability to allow transpiration processes through the wall’s perimeters. Plaster is a mortar composed of a binding part that incorporates sand with a selected particle size distribution, not greater than 2 mm. The sludge, to be used as plaster, must satisfy requirements related to thermal insulation, resistance to moisture, mechanical resistance and good injection. For this purpose, low-content metals sludge, derived from the Luserna stone flaming and cutting slabs, are to be reused as a substitute for the sands and fine particles, respectively, that are normally used to produce plasters. The laboratory tests carried out on the finished product, in accordance with European standards, are as follows: water absorption, specific density, flexural and compressive strength, before and after freeze and thaw cycles, pull out, salt crystallisation cycle resistance and thermal conductivity. Chemical and leaching tests were carried out to verify the possible release of heavy metals into the environment after installation. The product quality was demonstrated as the cement mortars, incorporating the metals, did not allow their release in nature. A sludge recovery, in an unaltered state, was provided to reduce any costs connected to a pre-treatment and to make recovery economically advantageous for the stone sector.

## 1. Introduction

Typically, stone quarries and stone processing plants are designed to extract and produce a marketable product with the maximum output and profit, but stone waste production and environmental management are a consequence of this process. One of the main options for managing rock processing waste, according to European Strategies on Best Available Technique (BAT) [1], is for land use, e.g., as aggregates or for restoration. The three factors: cost, environmental performance and risk of failure must be considered for the choice of the waste management method. The directive 2008/98/EC [2] defines the concept of “end-of-waste” and the priority order actions hierarchy for waste management. Prevention on products is a measure aimed at reducing the amount of waste and reducing the content of hazardous materials; re-use, recycling and recovery of waste are all actions aimed at resource efficiency, for reducing the environmental impact.

The amount of waste generated from sawing rock blocks into slabs, in Italy, is from 20% to 50% of the total slab production. In the Piedmont region, in 2016, the amount of sludge produced from the sawing process was about 22,756.30 tons, a lesser value compared to 2014, which amounted to 36,837.13 tons, this being due to the recent economic crisis in the sector [3]. The huge volume, the very fine particle size and the chemical characteristics of this material are the main problems related to its possible recovery.

The sawing sludge derived from the marble/limestone processing has been the subject of many studies, which has seen it as a component, able to partially replace cement in concrete, with a good performance in terms of mechanical and physical strength [4,5,6,7]. The correct use of marble/limestone sludge in concrete mixtures improves compaction, thanks to its very fine particle size distribution. Furthermore, its use as a substitute for cement generates a cost-saving (approximately 9%) and an energy-saving (approximately 1%) [6,8]. A study on limestone cutting waste, for use as mortars and plasters for building restoration, has shown that there is an economic and an aesthetic advantage [9].

The replacement of a specific amount of cement with quarry granite waste and sludge, derived from the silicate rock cutting process, is a more recent study. The partial replacement of cement, with sludge derived from the granite cutting process, increases the freeze and thaw resistance and the sulphates resistance, in terms of weight loss and mechanical strength, in the finished concrete products [10]. The addition of granite powder in mortar and concrete with different ratio has improved mortar and concrete compressive strengths and the concrete workability [11]. The waste deriving from granite stone polishing, used as a substitute in the concrete coarse aggregate, decreases the mechanical characteristics but increases the physical properties, such as water absorption, permeability and abrasion. The substitution of coarse aggregate with 20–30% of polished granite waste is recommended, but only for non-structural applications [12]. The replacement of fine aggregate with granite sludge shows an overall increase in strength [13], and the production of the denser matrix, yielding a 38% reduction in expansion and a 70% increase in chloride resistance without compromising workability and strength [14]. Silicate rock sludge containing a metals fraction, due to tool wear during the cutting process, could provide a change in mechanical characteristics and in the colour of the finished product. A study on sand replacement with powder, composed of iron and granite sludge, showed an increase of strength resistance [15]. Converting granite powder into reddish pigment, by means of calcination at 700–900 °C, produces benefits both in colour and in an acceptable compressive strength value [16]. The higher density of concrete and greater mechanical strength, due to the presence of granite sludge as a fine aggregate, produce durable concrete. This has been well documented by the improved response to various tests, such as carbonation, sulphate attack on abrasion, permeability and absorption of water [17].

## 2. Materials and Methods

### 2.1. Mix Design Components Characterisation

**Figure 1:**
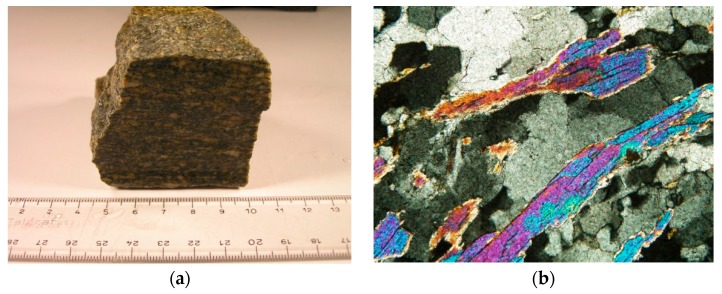
(**a**): macroscopic photo of Luserna stone; (**b**): microscopic photo of Luserna stone with its characteristic schistose texture due to white mica minerals orientation, K-feld: Potassic feldspar, Fn: Fengite, Bt: Biotite. The microscopic photo was carried out with optical microscope Leika in crossed Nicols, magnification 20×.

The processing waste of Luserna stone was used to prepare the design mix of the mortar. Luserna stone is a Piedmont gneiss characterised by a flat-schistose texture and by a heteroblastic structure with a micro-occhiadine tendency, due to the presence of millimetric micro-porphyroblasts (0.03–3 mm) (Figure 1). It is composed of Quartz (50%); Plagioclase (15%); Alkaline Feldspar (20%); White mica (5%); Chlorite (5%); Epidote (5%).

The two types of aggregate, derived from Luserna stone processing, were considered in this study:

- waste after flaming used as a sand aggregate.
- waste from sawing blocks into slabs through diamond tools, used as a filler aggregate.

**Figure 2:**
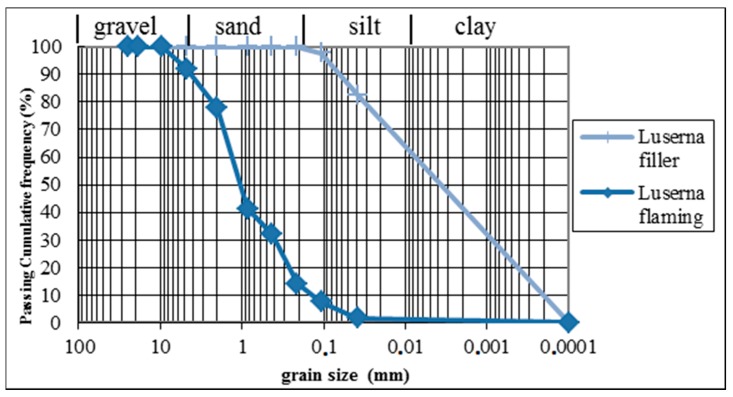
Particle size distribution analysis of two mix design components: Luserna filler from diamond blade sawing and Luserna sand from the flaming process.

A preliminary investigation was carried out for the particle size distribution analysis, performed according to UNI EN 933 Part 1 [18]. The results obtained are shown in Figure 2.

Luserna sand, from the flaming process, contains only mineral fractions that compose Luserna stone. Sawing sludge, from diamond blade cutting, contains a fraction from the mineral part and a metal fraction derived from the diamond tool’s wear. For this reason, magnetic separation, chemical analysis, leaching tests and XRPD (X-Ray Powder Diffraction) were performed on the Luserna sawing sludge sample. The quantification of the metal content in the sludge is necessary to define what is now a waste, a secondary raw material that can be used in other production processes (in accordance with Italian Legislative Decree 152/2006 [19], implementation of European Directive 2008/98/EC). Chemical analysis was performed, according to Italian Legislative Decree 152/2006, by means of Fkv Ethos Easy microwave mineraliser and ICP-Mass analyser. The results obtained, with the law threshold concentration limits, are reported in Table 1.

The results showed an excess of the concentration limits for the elements cobalt and Cr VI, but only for its reuse in the green area (Column A).

Leaching testing was performed according to UNI EN 12457-2 [20], taking into account the threshold limits concentration defined in the appendix A of UNI 10802 [21]. The results obtained are shown in Table 2.

On the basis of the chemical analysis, it could be asserted that Luserna sawing sludge could be re-cycled for plaster applications in buildings.

Magnetic separation, by means of Magnetic Separator (Eriez magnetics manufacturer, L Series, Model 4, Brasil) (Kolm-type high gradient), was performed to identify the quantity of metal content in the sludge sample. One of the main requirements for plaster application is thermal insulation. A low metal content can produce a lower thermal conductivity since metals are good thermal conductors. Table 3 shows the low percentage of magnetic (metals) fraction in the considered sludge sample.

The X-ray powder diffraction was performed with Rigaku model Geigerflex (model smartlab SE, Rigaku, Japan). The sample preparation was carried out by means of grinding, through a pestle, the sludge to obtain a fine powder (with dimensions between 25 and 10 m). This procedure is necessary to improve the homogeneity of the material.

**Figure 3:**
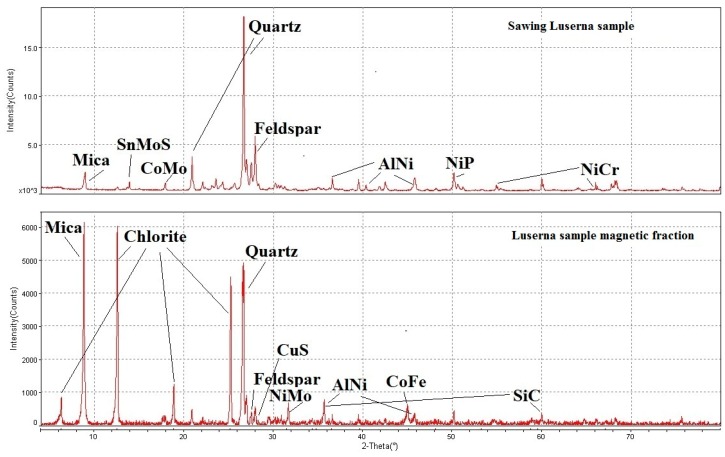
Comparison between the diamond blade sawing Luserna sample spectrum (over) and after magnetic separation (under).

Only the sample deriving from diamond blades sawing was analysed since the flaming sample has only components of the Luserna stone of which the composition is known. The analysis was carried out both on the sample as it is and on the sample after the magnetic separation on the magnetic fraction. This choice was due to the complexity in the interpretation of the results. The sample was composed of several minerals and several metals: for this reason, the peaks were overlapped. With magnetic separation, there was an elimination of some minerals phases, so the peaks of the metal were more evident. A comparison of spectrum for samples as it is and with the only magnetic fraction is shown in Figure 3.

The Luserna diamond blade sawing sample showed minerals like Quartz, Feldspar and Mica and some peaks of metals that compose both the steel core of the disc and the matrix of the diamond segments. The metals identified were: Cobalt Molybdenum Oxide (CoMoO_3_), Aluminum Nickel (Al_3_Ni), Nickel Phosphide (NiP) and Nickel Chromium Oxide (NiCrO_3_).

In the analysis performed on magnetic fraction, we could observe a peak related to the Chlorite and other metals that were not identified in sample analysis before magnetic separation. CuS, Cobalt Iron (Co_3_Fe_7_), Nickel Molybdenum Phosphide (NiMoP_2_) and Silicon Carbide (SiC) were identified. All elements were contained in high-speed tool steels.

### 2.2. Plaster Mix Design

The preparation of plaster mix design was carried out at TEK.SP.ED s.r.l. company (Casandrino, Napoli). The plaster types differ according to the kind of binder used. In this research, the binders were hydrated lime and portland cement, with a predominance of portland cement. The mix design involves the presence of lime, cement, aggregates, water, reinforcing fibres and foam. It is known from the literature [22] that the foam improves the thermal conductivity characteristics of the plaster but worsens the mechanical characteristics, such as compression. The foam, in this case, was used to give the final material a certain lightness, while the fibres gave greater mechanical resistance (tensile resistance). Table 4 shows the components of the mix design and the quantities used compared to 1 m^3^. Table 5 shows the mix design with the addition of Tufo’s powder (commercial ‘‘zeolitite”, rocks with zeolite content higher than 50 wt.%, coming from a quarry site located near Comiziano, Nola-Naples (Campanian Ignimbrite)), used mainly for chemical resistance test [23,24] compared to specimens produced with waste derived from the Luserna stone only. Adding of Tufo powder has been carried out since the Roman Empire period [25,26].

**Figure 4:**
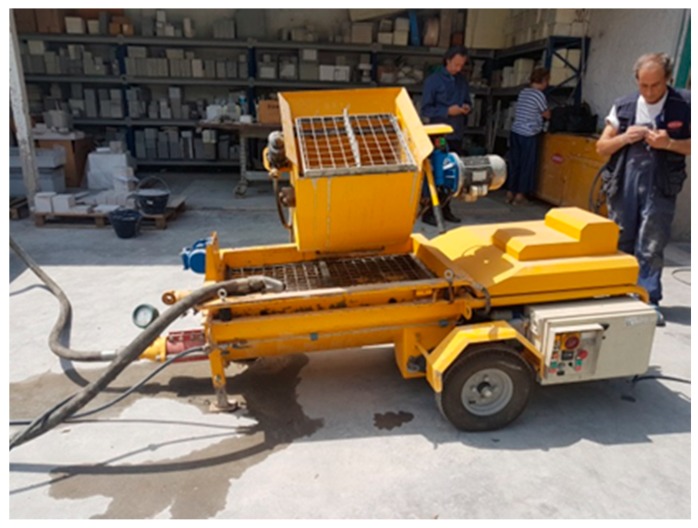
S8 EVM machinery creation of TEK.SP.ED s.r.l. used for eco-mortar for plaster mix design.

The components were mixed with the S8 EVM (single-phase electric with mixer) machinery (Figure 4) manufactured by TEK.SP.ED. s.r.l. company (Casandrino, Napoli, Italia). This machine is low power consumption, has a mixing system and a system for pumping plaster directly on site.

**Figure 5:**
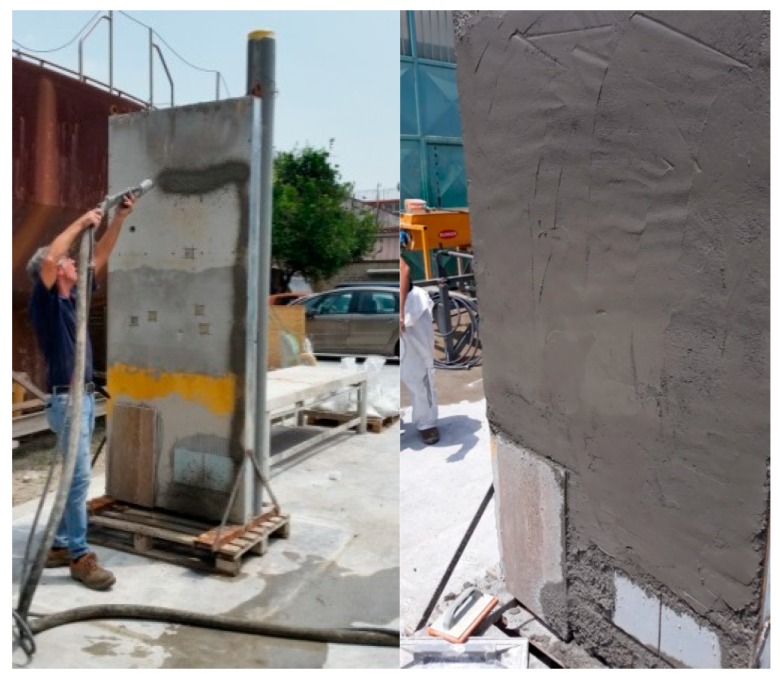
Left: Plaster pouring on a vertical panel. Right: results after pouring.

Once the mix design was prepared, the material was poured onto a vertical panel to simulate conventional industrial practice (Figure 5). In this way, both the adhesion of the material to the support and its behaviour with regards to hot summer temperatures could be tested. This critical weathering condition could cause cracks and fractures, on normal plasters, that could worsen over time.

**Figure 6:**
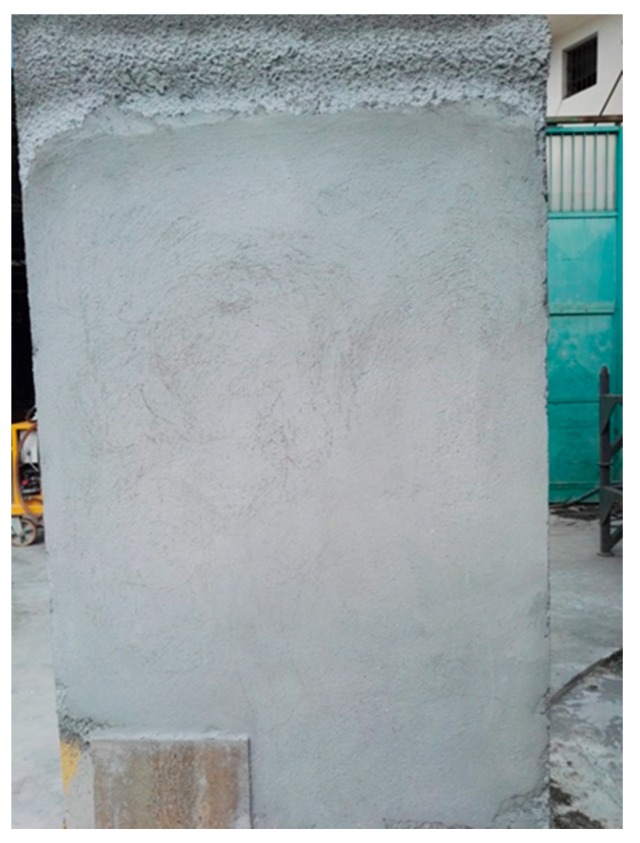
Pouring plaster results after two days from its application on the panel.

After two days, curing time (critical weathering condition), the results of plaster pouring were optimal. Figure 6 shows the homogeneity of the product obtained and the absence of fractures and cracks.

**Figure 7:**
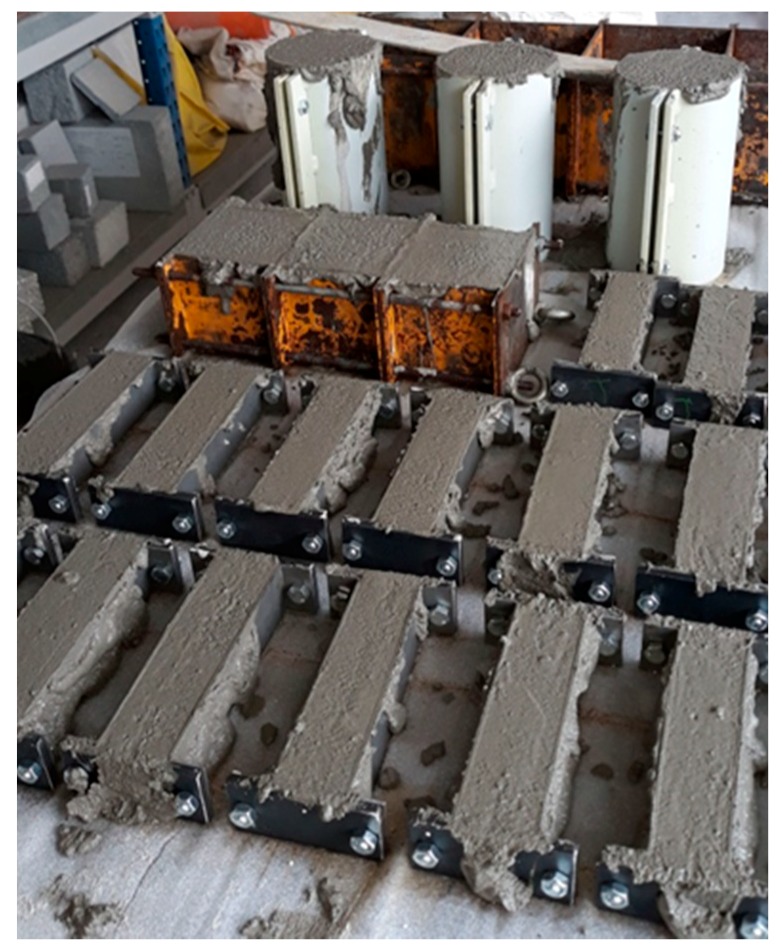
Plaster specimens prepared for physical-mechanical tests, according to standard UNI EN 998-1 and 2.

Using the same mix design, specimens were prepared for the physical-mechanical tests (Figure 7.). The number of specimens and their size were in accordance with the standards for each test reported in Table 6.

### 2.3. Physical Properties

The following laboratory tests were performed to investigate the physical properties of the plaster: bulk density, spreading test, thermal conductivity and water absorption.

#### 2.3.1. Bulk Density

Bulk density testing was carried out, weighing one cubic meter of material in the fresh state. For dry condition bulk density evaluation, three samples of plaster were placed in an oven at 105 °C. This was followed by two weighings, two hours apart, to verify that the mass was constant. Once the mass of the dry specimen was obtained (Ms, dry), the formula used to calculate the apparent bulk density for each specimen is as follows:

bulk density [kg/m^3^] = (Ms, dry)/Vs (1)

where Vs is the volume of the mold (m^3^).

The average value of the three measurements was then calculated, approximating the result to 10 kg/m^3^.

#### 2.3.2. Spreading Test

A spreading test was carried out using a Hagerman cone, according to ASTM D 6103 of 2017. The cone has a chamfer of 45°, an upper diameter of 70 mm and a bottom diameter of 100 mm.

#### 2.3.3. Thermal Conductivity

Thermal conductivity testing was performed using a KD2 Pro (Meter group manufacturer, Munich, Germany) with probe RK-1 model, 6 cm long and 3.9 mm diameter. Thermal conductivity was carried out on three samples, at 15 and 68 days of curing time and in dry condition (placed in the oven for 48 h at 60 °C).

#### 2.3.4. Water Absorption

Water absorption was carried out on 12 samples, with 6 samples not subjected to a freeze and thaw cycle, and the other 6 samples after a freeze and thaw cycle. The procedure involved placing specimens in an oven at 60 °C to obtain a constant mass. Once the dry weight (Md) of the specimens was obtained, the specimens were placed in water until saturation. Subsequently, the specimens were weighed to obtain the saturated mass (Ms). The results were expressed as the percentage of absorption obtained according to the equation:

WA[%] = (Ms − Md)/Md × 100
(2)

where Ms = saturated mass (g); Md = dry mass (g).

### 2.4. Mechanical Properties

The following laboratory tests were performed to investigate the mechanical properties of the plaster: flexural strength, compressive strength, pull out.

#### 2.4.1. Flexural Strength

Flexural strength was performed on three samples, according to UNI EN 1015-11 of 2007. A constant speed load of 50 N/s was applied to obtain a fracture between 30 s and 90 s. Flexural strength, after the freeze and thaw cycle, was performed according to UNI EN 12371 of 2010, which is related to natural stone, as it is not a test required by plaster standards. Twenty-five freeze and thaw cycles were performed with a four-hour duration of each cycle, consisting of two hours at −15 °C (in a saturated solution of sodium chloride) and two hours at 21 °C. The flexural test was performed seven days after the last cycle, according to UNI EN 1015-11:2007.

#### 2.4.2. Compressive Strength

Compressive strength was performed on six samples, according to UNI EN 1015-11 of 2007. Freeze and thaw cycle was performed, according to UNI EN 12371 of 2010, related to natural stone, as it is not a test required by plaster standard.

#### 2.4.3. Pull out

Pull out test was carried out, according to UNI EN 1015-12 of 2016, by TEK.SP.ED company on plaster pouring directly on the vertical panel after 28 days of curing.

### 2.5. Chemical Properties

The following laboratory tests were performed to investigate the chemical properties of the plaster: resistance to salt crystallisation, chemical analysis and leaching test.

#### 2.5.1. Resistance to Salt Crystallisation

Resistance to salt crystallisation was carried out on two samples with Luserna stone mix design (L1 and L2) and two samples of the mix with added Tufo Powder (T1 and T2), according to UNI EN 12,370 of 2001, which is related to ornamental stone standard, as it is not a test required by plaster standards. The test procedure included the use of a 14% solution of sodium sulphate decahydrate with the solution only being used for a single test cycle. The specimens were dried until a constant mass was attained, then weighed. The procedure was carried out for a total of 15 cycles. Each cycle involved the immersion of the specimens in the solution for two hours, at a temperature of 20 °C, and then drying in the oven at 105 °C for a time ranging from 10 to 15 h. After the 15th cycle, the specimens were placed in water for 24 h at 23 °C and then rinsed in water. They were then weighed, after drying to constant mass, to evaluate the mass lost during the test. The results were expressed as a percentage and obtained using the following formula:

∆M = (Mf − Md)/Md × 100
(3)

where Mf is the final mass (g); Md is the initial mass (g).

#### 2.5.2. Chemical Analysis and Leaching Test

Chemical analysis and leaching tests were performed according to D.Lgs. 152/2006. These tests are not required for plaster, but they were nevertheless performed to verify the content of the metals in the sludge after the recovery.

## 3. Results and Discussion

### 3.1. Physical Properties

The average values obtained are reported in Table 7.

**Figure 8:**
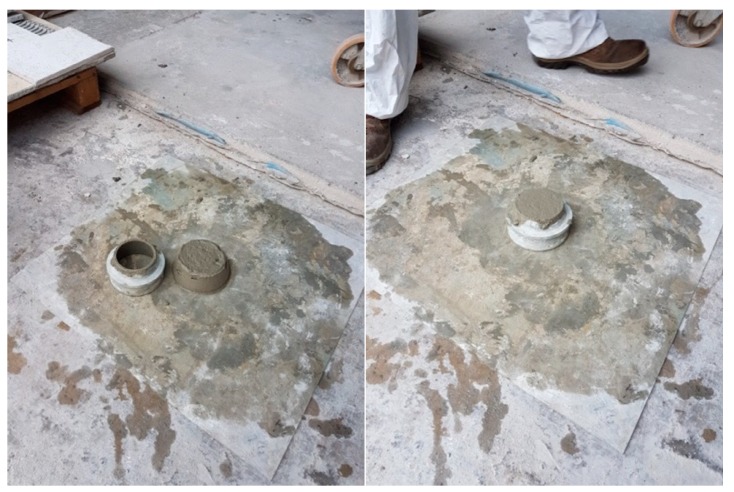
Spreading test results. No loss of shape is shown.

Plaster regulations state that plaster can be considered a light plaster if it has a dry density less than 1300 kg/m^3^. The eco-plaster can, therefore, be defined as a light plaster. Once the cone was removed, the plaster had not lost its shape (Figure 8).

**Figure 9:**
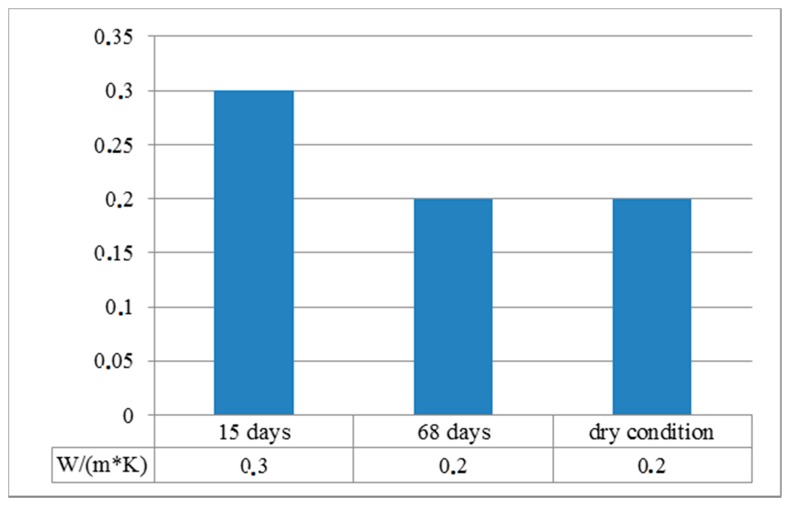
Thermal conductivity results of eco-plaster. Comparison at 15 days, 68 days and in dry condition.

Figure 9 shows the results obtained from the thermal conductivity tests. The values obtained indicated good thermal insulation of the plaster. Furthermore, the 68-day and dry condition values demonstrated the thermal stability of the mixture.

The results of water absorption tests showed that there was no change in the porosity of the specimens after the freeze and thaw cycles.

Comparing our plaster with some commercial lightened plasters [38,39], in terms of thermal conductivity, our plaster obtained better values: 0.302 (W/m·K) after 15 curing days and 0.201 (W/m·K) in dry condition, with respect to 0.54 (W/m·K) or 0.47 (W/m·K) of another product.

### 3.2. Mechanical Properties

The average values obtained by means of mechanical tests are shown in Table 8.

It could be seen that there was no appreciable difference between flexural strength values before and after the freeze and thaw cycle. The excellent results obtained placed the eco-plaster in the M10 compressive strength class (in accordance with UNI EN 998-2) and in the category of compressive strength CS IV (in accordance with UNI EN 998-1).

**Figure 10:**
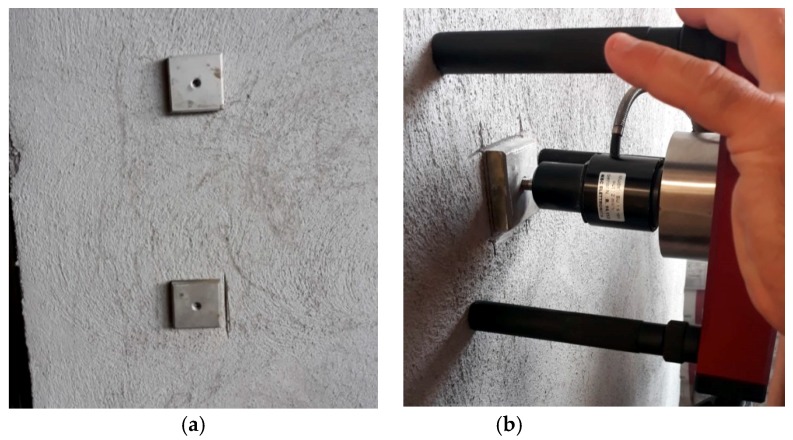
(**a**): Test plate mounted onto plaster; (**b**): Pull out test conducted.

**Figure 11:**
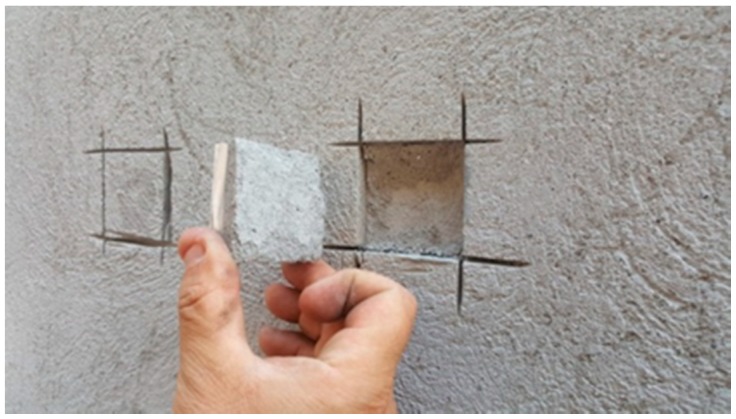
Test area to be patched back.

Figure 10 shows the test methodology for the pull-out test. Figure 11 shows the batched back test area and the good adhesion between plaster and panel support.

Performing the same comparison made for the physical properties on the mechanical properties [38,39], an improvement of the mechanical characteristics was observed both in terms of compressive and flexural strength and in terms of adhesion. Our plaster was in compressive strength category CS IV against the resistance class CS II of the other lightened plasters, with a compressive strength value of 11.97 MPa against the 4.4 MPa or 3MPa of other plasters. In terms of adhesion, our plaster obtained 1.55 MPa value against 0.3 MPa or 0.15 MPa of the others.

### 3.3. Chemical Properties

#### 3.3.1. Salt Crystallisation Resistance

Table 9 shows the results of the tests for the four specimens L1, L2, T1 and T2.

Samples did not show big weight and aesthetical variations after the salt cycle. Upon verification of the physical and mechanical characteristics, water absorption and flexural strength were carried out on specimens L1, L2, T1 and T2.

**Figure 12:**
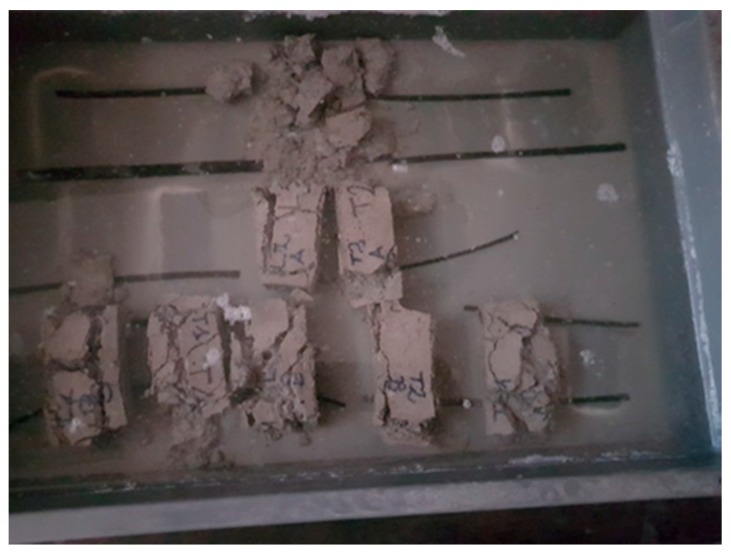
Imbibition of L1, L2, T1 and T2 specimens after salt crystallisation cycle and flexural strength test. Specimens breakdown.

Flexural strength test reported good values, higher than eco-plaster, before the salt cycle results (Table 10). While imbibition testing carried out after flexural strength caused the breaking of the specimens during the saturation step (Figure 12). This aspect is to be explored in the next study.

#### 3.3.2. Chemical Analysis and Leaching Test

The results of the two tests are shown in Table 11 for chemical analysis and Table 12 for leaching test.

All metal values were below the standard limits threshold. These chemical analysis results demonstrated the possibility of recovery of this type of sludge for plaster applications, instead of landfill disposal.

## 4. Conclusions

At present, there is no market, or regulation, in favour of the recovery of silicate sawing sludge for use as a plaster. This research aimed to contribute to the re-use of this “waste” as a “by-product”. The studied recoveries were foreseen on sludge without any treatment for economic advantages and to avoid landfill disposal. The recovery of sludge could turn a unidirectional system into a circular system.

Thermal-eco-mortar for plaster application, sawing sludge with low metal content from Luserna stone cutting with a diamond blade, improved the rheological, thermal and physical performance, conferring a light macroporous cellular structure by means of adding an organic foam. This characteristic facilitates the plaster installation, even for high thickness. The mechanical strength and thermal conductivity results exceeded the values of standard plaster, making this eco-friendly plaster an excellent product, ideal for use in an energy-saving role in buildings and in environments with high presence of humidity. Resistance to salt crystallisation showed a breakdown of samples after the water absorption test. This feature should be investigated by comparison with the results from a normal production plaster.

The first aim for future studies is to improve the mix design used for the plaster by increasing the amount of sludge used compared to the cement content. Furthermore, the use of sludge obtained immediately after the filter press process could be possible, taking into account the water content in this condition, to avoid a possible drying step in the oven, which would lead to high energy consumption. Finally, pozzolanic cement could be added to the mix design to avoid the disintegration of the plaster due to the action of the salts.

The circular economy approach, with the decrease in costs related to the disposal, the improvement of environmental conditions and the retrieval of, still exploitable, secondary raw materials must be necessary for an era in which the quantity of waste continues to increase. A collaboration between entities that produce waste and administrations, which provide guidelines for its recovery, is necessary to make what is now a unidirectional system, a circular system.

## Figures and Tables

**Table 1:** Results obtained from chemical analysis on Luserna sawing sludge tested (according to Lgs.D. 152/2006 Annex 5, title V, part IV and Art. 8 of the M.D. 05/02/1998). Last two rows indicate the limit of concentration of green area (column A) and commercial area (column B). * the results over the limits of column A.

Sample vs. Limits	Cu	Zn	Co	Ni	As	Cd	Total Cr	Cr VI	Pb	Hg	Fe
	mg/kg										
Luserna Sawing Sludge	41.89	24.82	26.15 *	<0.01	<0.1	<0.1	18.21	<5 *	23	<0.01	4340
Concentration Limit Column A	120	150	20	120	20	2	150	2	100	1	/
Concentration Limit Column B	600	1500	250	500	50	15	800	15	1000	5	/

**Table 2:** Results of the leaching test performed according to M.D. 05_02_1998, appendix A of UNI 10802 standard and UNI EN 12457-2 standard. Last row: threshold limit of metal concentration, by Italian regulation.

Sample vs. Limits	Ba	Cu	Zn	Be	Co	Ni	V	As	Cd	Total Cr	Pb	Se	Hg	NO_3_	F	SO_4_	Cl
	mg/l			µg/l										mg/l			
**Luserna Sawing Sludge**	7.4	<0.1	<0.031	<0.01	<1	<10	<1	<1	<0.1	<0.1	<1	<0.01	<1	<1	<0.1	1.3	1.86
**Threshold Limit**	1	0.05	3	10	250	10	250	50	5	50	50	10	1	50	1.5	250	100

**Table 3:** Results of magnetic separation on Luserna sawing sludge sample.

Sample	Magnetic Fraction (%)	Amagnetic Fraction (%)
Luserna Sawing Sludge	1.95	98.05

**Table 4:** Eco-mortar for plaster mix design with Luserna sawing sludge. Components and quantities for 1 m^3^. (*) filler from Luserna sawing sludge particle size distribution 0/300 µm; (**) polypropylene synthetic fibres = 6–12 mm.

Mix Design Components	M.U.	Quantities for 1 m^3^
Portland Cement 42.5R	kg	313
Lime Natural Hydraulic Lime 3.5	kg	38
Luserna Flaming Sand 0/3 mm	kg	813
Luserna Filler 0/0.1 mm (*)	kg	187
Micro Fibre (**)	kg	0.3
Natural Foam 72–75 g/m^3^	m^3^	0.37–0.38

**Table 5:** Eco-mortar for plaster mix design with the addition of Nola’s Tufo powder.

Mix Design Components	M.U.	Quantities for 1 m^3^
Portland Cement 42.5R	kg	313
Lime Natural Hydraulic Lime 3.5	kg	38
Luserna Flaming Sand 0/3 mm	kg	813
Nola’s Tufo Powder	kg	38
Luserna Filler 0/0.1 mm	kg	149
Micro Fibre	kg	0.3
Natural Foam 72–75 g/m^3^	m^3^	0.37–0.38

**Table 6:** Standardized f tests carried out on the eco-plaster product. n.d is not defined.

Tests Carried out	Standard References	Number of Specimens	Specimens Size
Bulk Density of Fresh Mortar	EN 1015-6:2007 [27]	1	A cubic meter of fresh mortar
Dry Bulk Density	EN 1015-10:2007 [28]	3	40 mm × 40 mm × 160 mm
Flexural and Compressive Strength	EN 1015-11:2007 [29]	3 (flexural strength) 6 (compressive strength)	40 mm × 40 mm × 160 mm (flexural strength) 40 mm × 40 mm × 80 mm (compressive strength)
Flexural and Compressive Strength after Freeze and Thaw Cycles	EN 12371:2010 [30]	3 (flexural strength) 6 (compressive strength)	40 mm × 40 mm × 160 mm (flexural strength) 40 mm × 40 mm × 80 mm (compressive strength)
Adhesive Strength–Pull Out	EN 1015-12:2016 [31]	4	n.d.
Compressive Strength Class	EN 998-2:2016 [32]	6	40 mm × 40 mm × 80 mm
Compressive Strength Category	EN 998-1:2016 [33]	6	40 mm × 40 mm × 80 mm
Spreading Test	ASTM D 6103:2017 [34]	3	Cone
Water Absorption	EN 13755:2008 [35]	12	40 mm × 40 mm × 160 mm
Thermal Conductivity at 15 and 68 Days of Curing	EN 1745:2012 [36]	3	Diameter 100 mm, height 200 mm
Thermal Conductivity in Dry Condition	EN 1745:2012 [36]	3	Diameter 100 mm, height 200 mm
Resistance to Salts Crystallisation	EN 12370:2001 [37]	4	40 mm × 40 mm × 160 mm
Chemical Analysis and Leaching Test	D.Lgs. 152/2006 Annex 5, Part IV and Art. 8 of D.M. 05/02/1998. [19]	1	n.d.

**Table 7:** Physical tests’ results on plaster.

Tests Carried out	Plaster
Fresh Condition–Density (kg/m^3^)	1380
Dry Condition–Density (kg/m^3^)	1264
Fresh Condition–Slump Test-Hargerman Cone Diameter: 100 mm	No evident slump
Thermal Conductivity-15 days (W/m·K)	0.302
Thermal Conductivity-68 days (W/m·K)	0.204
Thermal Conductivity–Dry Condition (W/m·K)	0.201
Water Absorption before Freeze and Thaw Cycle (%)	20
Water Absorption after Freeze and Thaw Cycle (%)	19

**Table 8:** Mechanical tests results performed on a plaster product.

Tests Carried out	Plaster
Pull off (MPa)	1.55
Compressive Strength (MPa)	11.97
Compressive Strength after Freeze and Thaw (MPa)	10.76
Compressive Strength Class (UNI EN998-2)	M10
Compressive Strength Category (UNI EN 998-1)	CS IV
Flexural Strength (MPa)	1.03
Flexural Strength after Freeze and Thaw (MPa)	0.93

**Table 9:** Results obtained from the resistance to salt crystallisation test.

Sample	M_d_ (g)	M_f_ (g)	ΔM (%)	Average Value (%)
**L1**	324.09	313.86	−3.16	−3.56
**L2**	351.09	337.2	−3.96	
**T1**	354.61	339.81	−4.17	−4.06
**T2**	355.8	341.72	−3.96	

**Table 10:** Flexural strength results after salt crystallisation cycle on specimens L1, L2, T1 and T2.

Samples	Flexural Strength (MPa)
**L1 and L2**	1.36
**T1 and T2**	1.99

**Table 11:** Chemical analysis test on eco-plaster. Last row: Standard limit concentration according to Lgs.D. 152/2006, Annex 5, title IV and Art. 8 of M.D. 05/02/1998.

Sample vs. Limit	Fe	Cu	Zn	Co	Ni	As	Cd	Cr	Pb	Cr VI	Hg
	mg/kg										
Plaster	7861	41	91	5	13	6	<1	15	10	<5	<5
Limit column B	/	600	1500	250	500	50	15	800	1000	15	5

**Table 12:** Leaching test on eco-plaster. Last row: Standard limit concentration according to Lgs.D. 152/2006, Annex 5, title IV.

Sample vs. Limit	Fe	Ba	Cu	Zn	Co	Ni	V	As	Cd	Cr	Pb	Be	Hg	Se
	mg/L				µg/L							mg/L		
Plaster	0.401	0.02	0.019	<0.01	<0.005	<0.005	<0.005	<0.01	<0.001	0.075	<0.01	<0.001	<0.0005	<0.025
Limits	/	1	0.05	3	250	10	250	50	5	50	50	10	1	10
